# Supplementary figures and images for: RXR-Mediated Remodeling of Transcriptional and Chromatin Landscapes in APP Mouse Brain: Insights from Integrated Single-Cell RNA and ATAC Profiling
Source: Cells. 2025 Dec 11;14(24):1970. doi: 10.3390/cells14241970 (PMC12732301; doi:10.3390/cells14241970)

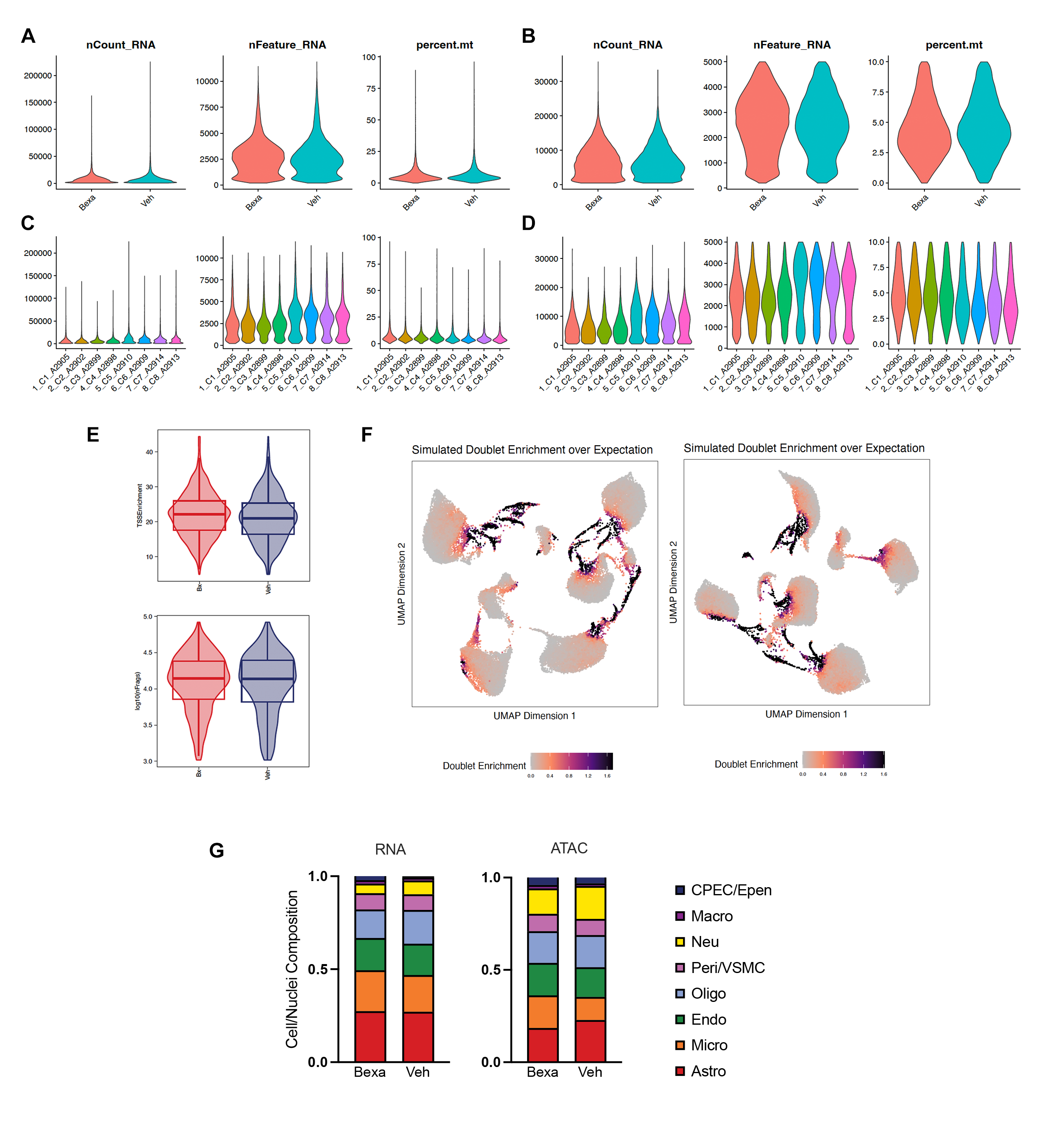

Supplement: Supplementary file 1 [file cells-14-01970-s001.zip › figure_S1_QC_Dec4.tif]

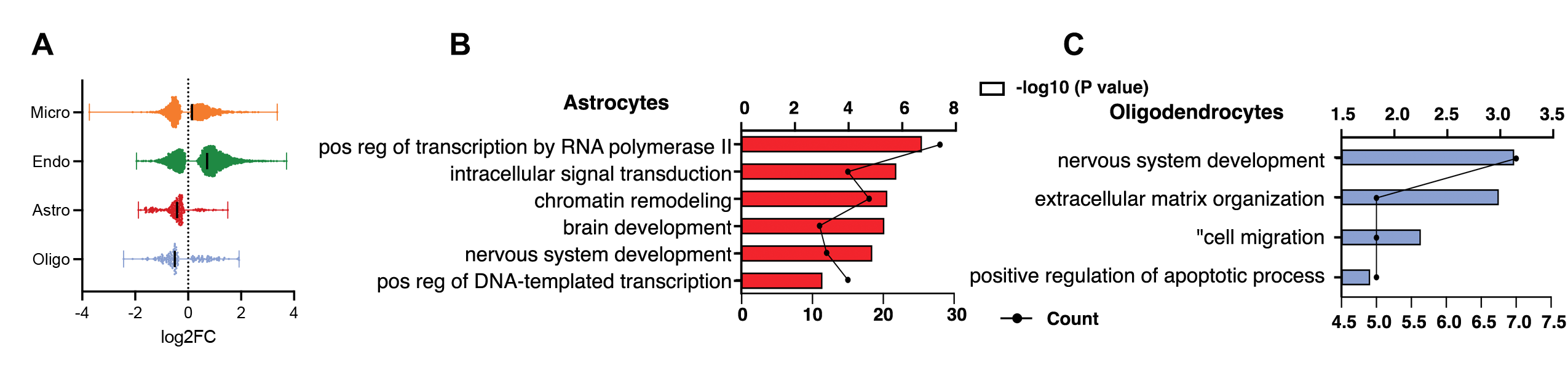

Supplement: Supplementary file 1 [file cells-14-01970-s001.zip › figure_S2-related to Fig 3-Dec5.tif]

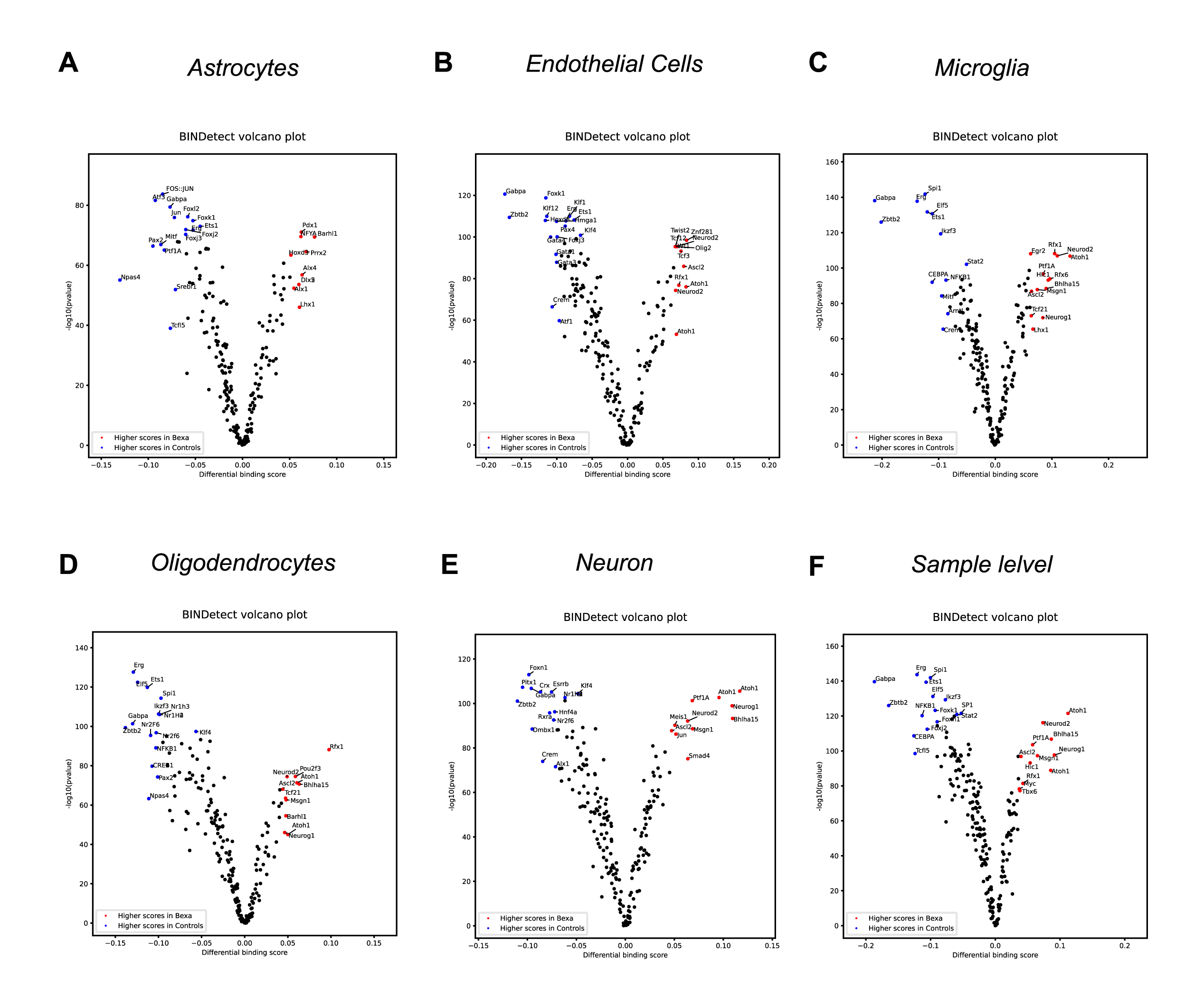

Supplement: Supplementary file 1 [file cells-14-01970-s001.zip › figure_S3_BINDetect_0209-related to Figure 5.tif]
